# Effect of smoking on subgingival microflora of patients with periodontitis in Japan

**DOI:** 10.1186/1472-6831-11-1

**Published:** 2011-01-05

**Authors:** Michiya Kubota, Mariko Tanno-Nakanishi, Satoru Yamada, Katsuji Okuda, Kazuyuki Ishihara

**Affiliations:** 1 Oral Health Science Center, Tokyo Dental College, Chiba 261-8502, Japan; 2 Department of Periodontology, Tokyo Dental College, Chiba 261-8502, Japan; 3 Department of Microbiology, Tokyo Dental College, Chiba 261-8502, Japan

## Abstract

**Background:**

Smoking is a risk factor for periodontitis. To clarify the contribution of smoking to periodontitis, it is essential to assess the relationship between smoking and the subgingival microflora. The aim of this study was to gain an insight into the influence of smoking on the microflora of Japanese patients with periodontitis.

**Methods:**

Sixty-seven Japanese patients with chronic periodontitis (19 to 83 years old, 23 women and 44 men) were enrolled in the present study. They consisted of 30 smokers and 37 non-smokers. Periodontal parameters including probing pocket depth (PPD) and bleeding on probing (BOP) and oral hygiene status were recorded. Detection of *Aggregatibacter actinomycetemcomitans, Porphyromonas gingivalis, Prevotella intermedia, Tannerella forsythia, Fusobacterium nucleatum/periodonticum, Treponema denticola *and *Campylobacter rectus *in subgingival plaque samples was performed by polymerase chain reaction. Association between the detection of periodontopathic bacteria and smoking status was analyzed by multiple logistic regression analysis and chi-square test.

**Results:**

A statistically significant association was found between having a PPD ≥ 4 mm and detection of *T. denticola, P. intermedia, T. forsythia*, or *C. rectus*, with odds ratios ranging from 2.17 to 3.54. A significant association was noted between BOP and the detection of *C. rectus *or *P. intermedia*, and smoking, with odds ratios ranging from 1.99 to 5.62. Prevalence of *C. rectus *was higher in smokers than non-smokers, whereas that of *A. actinomycetemcomitans *was lower in smokers.

**Conclusions:**

Within limits, the analysis of the subgingival microbial flora in smokers and non-smokers with chronic periodontitis suggests a relevant association between smoking and colonization by the specific periodontal pathogens including *C. rectus*.

## Background

Periodontitis, a local chronic inflammation in the supporting tissues of the teeth leading to progressive loss of periodontal ligament and bone, is believed to result from disruption of the homeostatic balance between periodontopathic bacteria and the host response to these microorganisms [1,2]. In addition, several factors, including smoking, socioeconomic status, behaviour and stress have been identified as potential risk factors for periodontitis [3].

Among these risk factors, smoking is strongly implicated in the development of periodontal disease. There is accumulating evidence for a higher level of periodontal disease among smokers [4,5]. Greater levels of clinical alveolar bone loss [5-7], tooth mobility [8], probing pocket depth [4,9] and tooth loss [10-12] have all been reported to be more severe in smokers than in non-smokers. Stoltenberg et al. [13] reported that the odds ratio for having a mean probing depth ≥ 3.5 mm was 5.3 times greater in smokers.

Mechanisms by which smoking affects the development of periodontitis are thought to be both direct and indirect. It has been suggested that modification of the periodontal microflora by smoking is involved in the development of periodontitis. It was shown that *in vitro *exposure of bacteria to cigarette smoking resulted in a marked decrease in the numbers of viable bacteria [14,15]. Zambon et al. [16] reported that smokers had significantly higher levels of, and were at greater risk of infection by *Tannerella forsythia*, in a study on 798 subjects with different smoking histories. Furthermore, they showed that smokers were 2.3 times more likely to harbour this periodontal pathogen than former smokers or non-smokers, after adjusting for disease severity. Umeda et al. [17] reported that current smokers displayed an increased risk (odds ratio, 4.6) for harbouring *Treponema denticola *in periodontal pockets, and that the presence of *Aggregatibacter actinomycetemcomitans, Porphyromonas gingivalis, Prevotella intermedia, Eikenella corrodens *or *Fusobacterium nucleatum *and smoking increased the risk of having a mean pocket depth of ≥ 3.5 mm. Likewise, Stoltenberg et al. [13] found that smoking was the strongest risk indicator for increased pocket depth. With regard to early onset periodontitis, Kamma et al [18] reported that smokers harboured higher levels of periodontal pathogens. On the other hand, other studies [13,19] found that both smokers and non-smokers exhibited similar subgingival microflora, suggesting that smoking has limited influence on the microflora involved in periodontal disease. Given these discrepancies, more information is needed to delineate the relationship between smoking and periodontal microflora. The aim of the present study was to gain insight into the influence of smoking on the microflora of Japanese patients with periodontitis.

## Methods

### Study Population

A total of 67 patients participated in the study. All were patients with a clinical diagnosis of chronic periodontitis, attending the Department of Periodontology, Tokyo Dental College, Chiba, Japan. The present study was conducted in accordance with the Helsinki Declaration, and informed consent was obtained from all participants before experiment. All procedures conformed to the protocols approved by the institutional ethical review board of Tokyo Dental College. The clinical diagnosis of chronic periodontitis was made on the basis of past dental history, clinical parameters and radiographic patterns of alveolar bone loss [20]. Exclusion criteria included previous periodontal treatment within the past year; systemic conditions that might affect periodontal disease activity or requiring premedication with antibiotics for periodontal probing; medications such as antibiotics, steroids, or non-steroidal, anti-inflammatory drugs within the past 6 months. Those with a diagnosis of aggressive periodontitis were also excluded. Smoking history was determined by means of a questionnaire. Based on the responses, the patients were classified as either smokers (current regular smokers) or non-smokers (who had never smoked tobacco). Smokers were those who smoke regularly for at least 6 months and had smoked more than 100 cigarettes in their lifetime. Former smokers were excluded.

### Clinical Examination

To determine periodontal status, probing pocket depth (PPD) and bleeding on probing (BOP) were examined at six sites (mesio-buccal, buccal, disto-buccal, disto-lingual, lingual and mesio-lingual surfaces) in each tooth, with the exception of the third molars. PPD was measured using a Williams probe from the gingival margin to the limit of the periodontal probe penetration at each site. Bleeding on probing (BOP) was recorded as the presence or absence of bleeding within 15 sec after probing. One trained examiner measured all the clinical indices. The presence or absence of supragingival plaque was recorded by O'Leary plaque control record [21].

### Detection of periodontopathic bacteria from subgingival plaque

A subgingival plaque sample was obtained as follows. After supragingival plaque was carefully removed and sampling sites were isolated with cotton rolls and air-dried, a subgingival plaque sample was collected with 2 sterile paper points per site. The plaque samples were collected from 3 to 4 sites with deepest PPD per patient. Each sample was subsequently transferred to 200 μl boiling buffer consisting of 20 mM Tris-HCl buffer (pH8.5), 2 mM EDTA and 1% triton X-100. The suspension was heated at 100°C for 10 min, and then centrifuged. Genomic DNA of the supernatant was obtained by phenol extraction. Periodontopathic microorganisms were detected by polymerase chain reaction (PCR). *P. gingivalis, P. intermedia *and *T. forsythia *were detected according to the method of Ashimoto et al [22], *Fusobacterium nucleatum/periodonticum *were detected according to the method of Kobayashi et al. [23] and *Treponema denticola*, *Campylobacter rectus *were detected by the method described by Yamazaki-Kubota et al [24]. Obtained PCR products were analyzed using 2% agarose gel electrophoresis. The relative intensity of the bands in the scanned image was compared to the control bands from known bacterial numbers (1 ×10^2 ^to 1 ×10^4 ^cells of each species) using the public domain Image J program (NIH; http://rsbweb.nih.gov/ij/), and categorized into four groups (0 to 3).

### Statistical analysis

The association between periodontitis (BOP positive or PPD ≥ 4 mm) and detection of bacteria was assessed using multiple logistic regression analyses adjusting for age, gender, oral hygiene and smoking status. For data analysis, each variable was coded as follows: Gender: male = 1, female = 0; Smoking: yes = 1, no = 0; Oral hygiene status: good (plaque score ≤ 30%) = 0, poor (plaque score ≥ 30%) = 1, Relative level of subgingival periodontal pathogens: (intensity of band < 10^2 ^cells) = 0, (10^2 ^~ 10^3 ^cells) = 1, (10^3 ^~10^4 ^cells) = 2 and (10^4 ^cells ≤) = 3 as described above. The relationship between detection of bacterial species and smoking status was determined by Pearson's chi-square analysis.

Computations were carried out with SAS Ver. 8.02 software. A probability value of < 0.05 was considered as statistically significant.

## Results

### Relationship between periodontal status and etiological factors

The demographic information, periodontal parameters and smoking status are summarized in Table 1. The total number of sites sampled for subgingival plaque was 135 for non-smokers and 118 for smokers. The seven periodontopathic bacteria were detected from 20% to 75% of the sites tested, with the detection rate for *P. gingivalis *being highest (75%). Table 2 shows the odds ratios for having a PPD of 4 mm or greater after adjusting for confounders. A statistically significant association (p < 0.05) was found between PPD ≥ 4 mm and detection of *T. denticola, P. intermedia, T. forsythia *or *C. rectus*, with odds ratio ranging from 2.17 to 3.54. In addition, males showed a higher risk (odds 3.09) for having a PPD ≥ 4 mm. The risk for having a PPD ≥ 4 mm in smokers was no different from that in non-smokers. In contrast, having BOP positive was significantly associated with being a smoker.

**Table 1:** Demographic information, periodontal and smoking status of participants

	Non-smoker (n = 37)	Smoker (n = 30)
Gender (M; F)	10; 27	13; 17
Age*	52.5 ± 15.6	46.2 ± 14.1
PPD (mm)*	5.01 ± 2.24	5.37 ± 2.62
Mean BOP + (% sites)	34.1	55.1
Cigarette consumption* (cig/day)	-	16.0 ± 7.2
Duration of smoking* (years)	-	21.1 ± 12.4

*mean ± standard deviation

PPD (probing pocke depth); BOP (Bleeding on probing)

**Table 2:** Adjusted odds ratio for probing pocket depth (PPD) ≥ 4 mm

Factor	Odds Ratio (95% CI)*	P-value
Age	0.991 (0.97 - 1.01)	0.450
Gender	3.09 (1.365 - 7.0)	0.007

*A. actinomycetemcomitans*	1.626 (0.72 - 3.69)	0.245
*P. intermedia*	2.790 (1.23 - 6.32)	0.014
*P. gingivalis*	0.710 (0.34 - 1.47)	0.364
*T. forsythia*	2.171 (1.03 - 4.58)	0.042
*F. nucleatum/periodonticum*	1.624 (0.84 - 3.16)	0.153
*T. denticola*	3.544 (1.48 - 8.52)	0.005
*C. rectus*	2.889 (1.22 - 6.85)	0.016

Smoking	0.637 (0.32 - 1.26)	0.194
Oral hygiene status	1.213 (0.57 - 2.57)	0.617

* 95% confidence interval, multiple logistic regression analysis

A significant association was noted between BOP and the detection rates of *C. rectus *or *P. intermedia*, and smoking, with odds ratios ranging from 1.99 to 5.62 (Table 3).

**Table 3:** Adjusted odds ratio for bleeding on probing (BOP) positive

Factor	Odds Ratio (95% CI)*	P-value
Age	0.987 (0.97 - 1.01)	0.238
Gender	1.545 (0.71 - 3.36)	3.356

*A. actinomycetemcomitans*	1.074 (0.50 - 2.30)	0.854
*P. intermedia*	2.214 (1.09 - 4.48)	0.027
*P. gingivalis*	0.642 (0.31 - 1.34)	0.235
*T. forsythia*	1.358 (0.62 - 2.96)	0.441
*F. nucleatum/periodonticum*	1.154 (0.61 - 2.18)	0.659
*T. denticola*	1.095 (0.52 - 2.31)	0.811
*C. rectus*	5.623 (2.71 - 11.65)	<.0001

Smoking	1.986 (1.07 - 3.69)	0.030
Oral hygiene status	1.281 (0.62 - 2.64)	0.502

* 95% confidence interval, multiple logistic regression analysis

### Relationship between smoking status and detection of periodontal pathogens

When the relationship between smoking status and detection of periodontal pathogens was sought by chi-square test, *A. actinomycetemcomitans *or *C. rectus *showed a significant association (Table 4). The odds ratio for colonization by *A. actinomycetemcomitans *in smokers was approximately 0.5, whereas that for *C. rectus *was 1.7 (p < 0.05).

**Table 4:** Periodontal pathogens with significant association with smoking

Species	Odds Ratio (95% CI)*	P-value
*A. actinomycetemcomitans*	0.4591 (0.26 - 0.80)	0.005
*C. rectus*	1.6875 (1.01 - 2.81)	0.043

* 95% confidence interval, chi-square test

## Discussion

In the present study, the detection of *T. denticola, C. rectus, T. forsythia *or *P. intermedia *was associated with having a PPD of 4 mm or greater. This finding was in line with the previous reports, which implicate these periodontal pathogens in the etiology of periodontitis [17,25-27]. Also in our study, the detection of *C. rectus *or *P. intermedia *increased at sites with PPD ≥ 4 mm or BOP positive. Takeuchi et al [28] reported that the detection of *C. rectus *was high in aggressive periodontitis lesions in Japanese. [29]. The detection of *C. rectus *in plaque samples was significantly correlated with clinical parameters such as PPD, BOP, and Gingival index in Japanese subjects [30]. Increased proportions of *P. intermedia *and *C. rectus *have been associated with both the initiation and progression of the disease in other populations as well [31,32]. Alves et al [33] reported that an increase in probing depth correlated with the number of genotypes of *P. intermedia*. These reports suggested that *P. intermedia *and *C. rectus *are associated with advanced periodontitis.

In this study, the colonization by *C. rectus *was significantly higher in smokers, while that by *A. actinomycetemcomitans *was lower. This finding of *C. rectus *in smokers agrees with that of a previous report by Kamma et al [18]. They reported that *P. gingivalis, T. forsythia, Parvimonas micra *and *C. rectus *were predominant in subgingival microflora in smokers, but were not so in non-smokers with early-onset periodontitis. It is important, however, to note that our results might be affected by differences in PPD or BOP between smokers and non-smokers, because of the statistical method we used.

The presence of *C. rectus *was reported to be correlated with the presence of *P. gingivalis*, *T. denticola *or *T. forsythia *[34]. *C. rectus *utilizes end products of other microorganisms such as formate from streptococci and H_2 _from Prevotella and Porphyromonas [35]. Our finding that the prevalence of *C. rectus *and *P. intermedia *increased at sites with PPD ≥ 4 mm or BOP positive, suggested that the change in microflora induced by smoking would provide a certain advantage to colonization by a subset of pathogens including *C. rectus*. However, further analysis is required. Although *C. rectus *and *A. actinomycetemcomitans *are facultative anaerobes, *C. rectus *is asaccharolytic and negative in many routine biochemical tests, while *A. actinomycetemcomitans *is saccharolytic [36]. It is possible that these differences in metabolism affect colonization by *A. actinomycetemcomitans*.

Several reports demonstrated limited influence of smoking on periodontal pathogens [19,37,38]. Among them, Bostrom et al [37] found that the detection rate of periodontopathic bacteria was higher in smokers, although not significantly. Haffajee et al [39] reported that species such as *T. forsythia, P. gingivalis, T. denticola, P. intermedia, P. micra *and *P. nigrescens *were significantly higher in the current smokers at sites with pocket depth < 4 mm compared with the non-smokers. Recently, Teixeira et al [40] reported that *P. gingivalis *with type IV fimbriae was associated with disease severity of smokers. Further cohort analysis between colonization of periodontopathic bacteria and smoking is required to clarify the whole situation.

In our study, the risk for having a PPD of 4 mm or greater in smokers was no different from that in non-smokers (odds ratio for smoking; 0.637). It has been reported that PPD in smokers was higher than that in non-smokers [4], and being a moderate/heavy smoker was a risk indicator for PPD ≥ 6 mm (odds ratio = 3.7, 95% confidence intervals: 1.4-10.1) [41]. In this study, the mean cigarette consumption per day was 16.0. Twenty seven of 30 patients in the present study can be categorized as light smokers in Corraini's study [41]. In their study, being a light smoker was not a risk indicator at p < 0.05. It is possible that the level of cigarette consumption affects the odds ratio, although further analysis after adjusting for deposition of plaque and frequency of smoking is required to clarify this discrepancy.

We found that the odds ratio for BOP-positive was significantly higher in smokers, although the risk of having a PPD of 4 mm or greater was not associated with smoking. Several studies have reported that BOP in smokers was at the same level or lower than that in non-smokers [42,43]. It was also reported that no difference was observed in BOP or gingival bleeding between non-smokers and smokers [44,45]. Although BOP indicates presence of inflammation, its sensitivity was reported as only 29% [46]. The difference in the relationship between BOP and smoking found in this and earlier studies might be explained by differences in the condition of periodontitis.

The relevant weakness of the present study is the relatively small sample size. Exact age and gender matching of smokers and non-smokers was not possible. Finally we could not obtain clinical attachment level data, mainly because of the limited time allowed for the single-appointment clinical examination. However, our results add to the existing literature by suggesting the salient relationship between the detection of periodontal pathogens and smoking status in a population of periodontitis patients in Japan. Further studies utilizing more comprehensive clinical assessment and quantitative microbiological method such as real-time PCR are necessary.

## Conclusions

Within limits, our data suggest that smoking favours colonization by the specific periodontopathic bacteria including *C. rectus*, and that this contributes to the disease severity in smokers.

## Abbreviations

PPD: probing pocket depth; BOP: bleeding on probing; PCR: polymerase chain reaction;

## Competing interests

The authors declare that they have no competing interests.

## Authors' contributions

MK carried out detection of the periodontopathic bacteria and drafted the manuscript. MTN carried out the subgingival plaque sampling and clinical examination. SY participated in the design of the study. KO carried out statitical analysis. KI conceiveed of the study and participated in the design, coordination and assisted to draft the manuscript. All authors read and approved the final manuscript.

## Pre-publication history

The pre-publication history for this paper can be accessed here:

http://www.biomedcentral.com/1472-6831/11/1/prepub

## References

[B1] Haffajee AD Socransky SS Smith C Dibart S Microbial risk indicators for periodontal attachment loss J Periodontal Res 1991 26 3 Pt 2 293 296 10.1111/j.1600-0765.1991.tb01662.x 1831856

[B2] Genco RJ Host responses in periodontal diseases: current concepts J Periodontol 1992 63 4 Suppl 338 355 10.1902/jop.1992.63.4s.338 1573548

[B3] Albandar JM Global risk factors and risk indicators for periodontal diseases Periodontol 2000 2002 29 177 206 10.1034/j.1600-0757.2002.290109.x 12102708

[B4] Bergstrom J Cigarette smoking as risk factor in chronic periodontal disease Community Dent Oral Epidemiol 1989 17 5 245 247 10.1111/j.1600-0528.1989.tb00626.x 2791514

[B5] Feldman RS Bravacos JS Rose CL Association between smoking different tobacco products and periodontal disease indexes J Periodontol 1983 54 8 481 487 6578319 10.1902/jop.1983.54.8.481

[B6] Bergstrom J Eliasson S Preber H Cigarette smoking and periodontal bone loss J Periodontol 1991 62 4 242 246 2037954 10.1902/jop.1991.62.4.242

[B7] Bolin A Lavstedt S Frithiof L Henrikson CO Proximal alveolar bone loss in a longitudinal radiographic investigation. IV. Smoking and some other factors influencing the progress in individuals with at least 20 remaining teeth Acta Odontol Scand 1986 44 5 263 269 10.3109/00016358609004732 3468736

[B8] Feldman RS Alman JE Chauncey HH Periodontal disease indexes and tobacco smoking in healthy aging men Gerodontics 1987 3 1 43 46 3471618

[B9] Goultschin J Cohen HD Donchin M Brayer L Soskolne WA Association of smoking with periodontal treatment needs J Periodontol 1990 61 6 364 367 2366143 10.1902/jop.1990.61.6.364

[B10] Ahlqwist M Bengtsson C Hollender L Lapidus L Osterberg T Smoking habits and tooth loss in Swedish women Community Dent Oral Epidemiol 1989 17 3 144 147 10.1111/j.1600-0528.1989.tb00009.x 2786792

[B11] Bergstrom J Floderus-Myrhed B Co-twin control study of the relationship between smoking and some periodontal disease factors Community Dent Oral Epidemiol 1983 11 2 113 116 10.1111/j.1600-0528.1983.tb01367.x 6573237

[B12] Osterberg T Mellstrom D Tobacco smoking: a major risk factor for loss of teeth in three 70-year-old cohorts Community Dent Oral Epidemiol 1986 14 6 367 370 10.1111/j.1600-0528.1986.tb01093.x 3466768

[B13] Stoltenberg JL Osborn JB Pihlstrom BL Herzberg MC Aeppli DM Wolff LF Fischer GE Association between cigarette smoking, bacterial pathogens, and periodontal status J Periodontol 1993 64 12 1225 1230 10.1902/jop.1993.64.12.1225 8106950

[B14] Ertel A Eng R Smith SM The differential effect of cigarette smoke on the growth of bacteria found in humans Chest 1991 100 3 628 630 10.1378/chest.100.3.628 1889244

[B15] Bardell D Viability of six species of normal oropharyngeal bacteria after exposure to cigarette smoke in vitro Microbios 1981 32 127 7 13 7339446

[B16] Zambon JJ Grossi SG Machtei EE Ho AW Dunford R Genco RJ Cigarette smoking increases the risk for subgingival infection with periodontal pathogens J Periodontol 1996 67 10 Suppl 1050 1054 8910822 10.1902/jop.1996.67.10s.1050

[B17] Umeda M Chen C Bakker I Contreras A Morrison JL Slots J Risk indicators for harboring periodontal pathogens J Periodontol 1998 69 10 1111 1118 9802709 10.1902/jop.1998.69.10.1111

[B18] Kamma JJ Nakou M Baehni PC Clinical and microbiological characteristics of smokers with early onset periodontitis J Periodontal Res 1999 34 1 25 33 10.1111/j.1600-0765.1999.tb02218.x 10086883

[B19] Preber H Bergstrom J Linder LE Occurrence of periopathogens in smoker and non-smoker patients J Clin Periodontol 1992 19 9 Pt 1 667 671 10.1111/j.1600-051X.1992.tb01716.x 1331203

[B20] Armitage GC Development of a classification system for periodontal diseases and conditions Ann Periodontol 1999 4 1 1 6 10.1902/annals.1999.4.1.1 10863370

[B21] O'Leary TJ Drake RB Naylor JE The plaque control record J Periodontol 1972 43 1 38 4500182 10.1902/jop.1972.43.1.38

[B22] Ashimoto A Chen C Bakker I Slots J Polymerase chain reaction detection of 8 putative periodontal pathogens in subgingival plaque of gingivitis and advanced periodontitis lesions Oral Microbiol Immunol 1996 11 4 266 273 10.1111/j.1399-302X.1996.tb00180.x 9002880

[B23] Kobayashi N Ishihara K Sugihara N Kusumoto M Yakushiji M Okuda K Colonization pattern of periodontal bacteria in Japanese children and their mothers J Periodontal Res 2008 43 2 156 161 10.1111/j.1600-0765.2007.01005.x 18302616

[B24] Yamazaki-Kubota T Miyamoto M Sano Y Kusumoto M Yonezu T Sugita K Okuda K Yakushiji M Ishihara K Analysis of matrix metalloproteinase (MMP-8 and MMP-2) activity in gingival crevicular fluid from children with Down's syndrome J Periodontal Res 2010 45 2 170 176 10.1111/j.1600-0765.2009.01214.x 19778333

[B25] Socransky SS Haffajee AD Cugini MA Smith C Kent RLJ Microbial complexes in subgingival plaque J Clin Periodontol 1998 25 2 134 144 10.1111/j.1600-051X.1998.tb02419.x 9495612

[B26] Mombelli A Casagni F Madianos PN Can presence or absence of periodontal pathogens distinguish between subjects with chronic and aggressive periodontitis? A systematic review J Clin Periodontol 2002 29 Suppl 3 10 21 discussion 37-38 10.1034/j.1600-051X.29.s3.1.x 12787203

[B27] Grossi SG Zambon JJ Ho AW Koch G Dunford RG Machtei EE Norderyd OM Genco RJ Assessment of risk for periodontal disease. I. Risk indicators for attachment loss J Periodontol 1994 65 3 260 267 10.1902/jop.1994.65.3.260 8164120

[B28] Takeuchi Y Umeda M Ishizuka M Huang Y Ishikawa I Prevalence of periodontopathic bacteria in aggressive periodontitis patients in a Japanese population J Periodontol 2003 74 10 1460 1469 10.1902/jop.2003.74.10.1460 14653392

[B29] Suda R Kobayashi M Nanba R Iwamaru M Hayashi Y Lai CH Hasegawa K Possible periodontal pathogens associated with clinical symptoms of periodontal disease in Japanese high school students J Periodontol 2004 75 8 1084 1089 10.1902/jop.2004.75.8.1084 15455735

[B30] Ihara H Miura T Kato T Ishihara K Nakagawa T Yamada S Okuda K Detection of *Campylobacter rectus *in periodontitis sites by monoclonal antibodies J Periodontal Res 2003 38 1 64 72 10.1034/j.1600-0765.2003.01627.x 12558939

[B31] Zambon JJ Reynolds HS Slots J Black-pigmented Bacteroides spp. in the human oral cavity Infect Immun 1981 32 1 198 203 6111541 10.1128/iai.32.1.198-203.1981 PMC350607

[B32] Ashley FP Gallagher J Wilson RF The occurrence of *Actinobacillus actinomycetemcomitans, Bacteroides gingivalis, Bacteroides intermedius *and spirochaetes in the subgingival microflora in relation to the early onset of periodontitis in a group of adolescents Oral Microbiol Immunol 1989 4 4 236 238 10.1111/j.1399-302X.1989.tb00260.x 2640319

[B33] Alves AC Napimoga MH Klein MI Hofling JF Goncalves RB Increase in probing depth is correlated with a higher number of *Prevotella intermedia *genotypes J Periodontol 2006 77 1 61 66 10.1902/jop.2006.77.1.61 16579704

[B34] Miyamoto E Nakano K Fujita K Nomura R Okawa R Matsumoto M Ooshima T Bacterial profiles of oral streptococcal and periodontal bacterial species in saliva specimens from Japanese subjects Arch Oral Biol 2009 54 4 374 379 10.1016/j.archoralbio.2009.01.010 19230860

[B35] Marsh PD Kuramitsu HK, Ellen RP Oral ecology and its impact on oral microbial diversity Oral Bacterial Ecology 2000 Wymondham: Horizon Scientific Press 11 65

[B36] Tanner A Lai C-H Maiden M Slots J, Taubman MA Characteristics of oral gram-negative species Contemporary Oral Microbiology and Immunology 1992 St. Louis: Mosby-Year Book, Inc 299 341

[B37] Boström L Bergström J Dahlén G Linder LE Smoking and subgingival microflora in periodontal disease J Clin Periodontol 2001 28 3 212 219 11284533 10.1034/j.1600-051x.2001.028003212.x

[B38] Darby IB Hodge PJ Riggio MP Kinane DF Microbial comparison of smoker and non-smoker adult and early-onset periodontitis patients by polymerase chain reaction J Clin Periodontol 2000 27 6 417 424 10.1034/j.1600-051x.2000.027006417.x 10883871

[B39] Haffajee AD Socransky SS Relationship of cigarette smoking to the subgingival microbiota J Clin Periodontol 2001 28 5 377 388 10.1034/j.1600-051x.2001.028005377.x 11350499

[B40] Teixeira SR Mattarazo F Feres M Figueiredo LC de Faveri M Simionato MR Mayer MP Quantification of *Porphyromonas gingivalis *and *fimA *genotypes in smoker chronic periodontitis J Clin Periodontol 2009 36 6 482 487 10.1111/j.1600-051X.2009.01411.x 19508247

[B41] Corraini P Baelum V Pannuti CM Pustiglioni AN Romito GA Pustiglioni FE Risk indicators for increased probing depth in an isolated population in Brazil J Periodontol 2008 79 9 1726 1734 10.1902/jop.2008.070586 18771375

[B42] Shimazaki Y Saito T Kiyohara Y Kato I Kubo M Iida M Yamashita Y The influence of current and former smoking on gingival bleeding: the Hisayama study J Periodontol 2006 77 8 1430 1435 10.1902/jop.2006.050422 16881812

[B43] Calsina G Ramon JM Echeverria JJ Effects of smoking on periodontal tissues J Clin Periodontol 2002 29 8 771 776 10.1034/j.1600-051X.2002.290815.x 12390575

[B44] Bergstrom J Eliasson S Dock J A 10-year prospective study of tobacco smoking and periodontal health J Periodontol 2000 71 8 1338 1347 10.1902/jop.2000.71.8.1338 10972650

[B45] van der Weijden GA de Slegte C Timmerman MF van der Velden U Periodontitis in smokers and non-smokers: intra-oral distribution of pockets J Clin Periodontol 2001 28 10 955 960 10.1034/j.1600-051x.2001.028010955.x 11686814

[B46] Lang NP Adler R Joss A Nyman S Absence of bleeding on probing. An indicator of periodontal stability J Clin Periodontol 1990 17 10 714 721 10.1111/j.1600-051X.1990.tb01059.x 2262585

